# Outcome of Wingspan Stent Using Aggressive Post-stent Balloon Dilation for Intracranial Atherosclerosis Stenosis

**DOI:** 10.3389/fneur.2021.757175

**Published:** 2021-10-25

**Authors:** Pang-Shuo Perng, Yuan-Ting Sun, Hao-Kuang Wang, Yu-Hsiang Shih, Jung-Shun Lee, Liang-Chao Wang, Chih-Yuan Huang

**Affiliations:** ^1^Division of Neurosurgery, Department of Surgery, National Cheng Kung University Hospital, College of Medicine, National Cheng Kung University, Tainan, Taiwan; ^2^Department of Neurology, National Cheng Kung University Hospital, College of Medicine, National Cheng Kung University, Tainan, Taiwan; ^3^Advanced Optoelectronic Technology Center, National Cheng Kung University, Tainan, Taiwan; ^4^School of Medicine for International Students, I-Shou University, Kaohsiung, Taiwan; ^5^Department of Neurosurgery, E-Da Hospital, I-Shou University, Kaohsiung, Taiwan; ^6^Department of Radiology, National Cheng Kung University Hospital, College of Medicine, National Cheng Kung University, Tainan, Taiwan; ^7^Department of Cell Biology and Anatomy, College of Medicine, National Cheng Kung University, Tainan, Taiwan; ^8^Institute of Basic Medical Sciences, College of Medicine, National Cheng Kung University, Tainan, Taiwan

**Keywords:** intracranial atherosclerosis disease, balloon, stent, wingspan, post-dilation

## Abstract

**Background:** Wingspan stent has gained interest for better long-term outcomes for intracranial atherosclerosis disease (ICAD). However, in-stent restenosis still presents as a problem and may cause postoperative neurological events. We aimed to find a way to prevent in-stent restenosis.

**Method:** Patients with stenosis >70% ICAD were treated with wingspan stent and were retrospectively reviewed. The patients were separated into two groups: one with post-dilation and the other without post-dilation. The outcomes of wingspan stenting were compared immediately after the surgery and at a 1-year follow-up.

**Results:** Overall, 28 patients were included for analysis, with 15 patients undergoing post-dilation and 13 patients not undergoing the procedure. The extent of stenosis was significantly lower in the post-dilation group than in the no post-dilation group, both immediately after the surgery (14.8 ± 10.2 vs. 28.5 ± 14.5%, *p* < 0.01) and at 1-year follow-up (25.8 ± 18.0 vs. 50.1 ± 23.2%, *p* < 0.01). The post-dilation method immediately expanded the stent diameter (2.89 ± 0.48 vs. 3.05 ± 0.44 mm, *p* < 0.001), and the diameter still increased at 1-year follow-up (3.05 ± 0.44 vs. 3.12 ± 0.43 mm, *p* < 0.01) due to the self-expandable property of the wingspan. Similarly, in the no post-dilation group, the stent size was also increased (2.70 ± 0.67 vs. 2.80 ± 0.64 mm*, p* < 0.01). However, at 1-year follow up, the luminal diameter was stationary in the post-dilation group (2.36 ± 0.73 vs. 2.46 ± 0.82 mm, *p* = 0.88) and decreased in the no post-dilation group (2.24 ± 0.56 vs. 1.60 ± 0.79 mm, *p* < 0.01). The periprocedural complication rate was similar between the groups.

**Conclusion:** The post-dilation method can be feasibly performed and can offer better stent expansion and apposition in the wingspan system. By applying this technique, we might prevent in-stent restenosis and improve neurological outcomes.

## Introduction

Intracranial atherosclerosis disease (ICAD) accounts for nearly 10% of the stroke and transient ischemic attack (TIA) cases worldwide (1) and up to 50% of the cerebral vascular events in the Chinese population (2). With the progress in image modalities, significant improvement has been made in the diagnosis and management of ICAD (3). Current treatment strategies for ICAD include medical treatment combined with aggressive lifestyle management and endovascular revascularization. Several clinical trials have evaluated the effect of medical treatment in comparison to percutaneous transluminal angioplasty and stenting (PTAS) on the prevention of stroke, including SAMPPRIS and VISSIT. The results showed a higher 30-day stroke or death rate in the PTAS group (14.7–24.1%) than in the medical group (5.8–9.4%) (4, 5). The aggressive medical treatment seemed to reduce 1-year stroke or death rate (12.2–15.1%) when compared with the WASID trial (21.8–22.1%) (6). However, with improvements in surgery technique, PTAS had resurfaced after the WEAVE trial, with its conclusion stating that with careful patient selection criteria and adequate surgeon experience, the periprocedural stroke and death rate were lower than expected (2.6%) (7). Wingspan stenting system (Stryker) is a self-expandable stent and the only stenting system approved by the Food and Drug Administration for ICAD (8). With the ongoing CASSISS trial (9), wingspan has the potential to become one of the solutions for ICAD.

However, the wingspan system is not totally without consequences. In-stent restenosis (ISR) after stent placement has been an important factor in recurrent stroke or TIA (10). Though mostly asymptomatic, ISR can reach up to 30%, with an incidence that is much higher than expected (11, 12). The treatment of ISR can be challenging due to the design of the stent system and relatively low metal surface area coverage (12). Therefore, we seek an innovative technique to reduce the extent of the ISR. Post-dilation had been used in stents for carotid artery and coronary artery in selected patients (13, 14), but its effect on the wingspan system has yet to be found. We aimed to determine if post-dilation impacts postoperative ISR rate and the stent and lumen diameters for ICAD patients.

## Materials and Methods

### Patient Selection

Information of all patients who underwent wingspan stenting was retrospectively reviewed from one Medical Center between December 2018 and March 2021. The inclusion criteria for wingspan stenting included (1) ICAD with the extent of stenosis >70% using the WASID measurement method (15), (2) the stenosis lesion corresponded to the neurologic events, and (3) the interval from the final stroke or TIA to the surgery was at least 3 weeks (16). The excluding criteria included: (1) age <18 years, (2) artery stenosis combined with the non-atherosclerotic lesion, such as arteriovenous malformation and brain tumors, and (3) intolerance to antithrombotic therapy or procedure-related general anesthesia, heparinization, and contrast agents. The patients received no post-dilation stenting procedure until December 2019 and with post-dilation stenting from January 2020. This study was approved by the institutional review board of our institute (B-ER-110-084). Individual patient consent was not required by the review board and therefore not sought.

### Perioperative Management

Aspirin (100 mg per day) and clopidogrel (75 mg per day) were administered orally at least 7 days before the procedure. Exceptions were made for patients already taking antithrombotic agents like non-vitamin K antagonist oral anticoagulants or cilostazol. In such patients, only aspirin was added. After the procedure, clopidogrel was continued for at least 12 months, and aspirin was continued lifelong. Heparinization with a 75-U/kg dosage bolus was administered before the surgery, followed by enoxaparin 30 IU/kg (Sanofi, France) administered after the surgery for 1 day.

### Surgical Technique

Each patient underwent three-dimensional rotational angiography. The proper working view was carefully reviewed and decided before the surgery. The procedure was performed using a biplane angiographic system (Artis zee Biplane; Siemens, Germany) or single-arm angiosuite (Artis Zeego; Siemens, Germany) under general anesthesia. A 6-French guiding sheath (Flexor Shuttle; Cook, USA) was inserted either in the right or left femoral artery to reach the common carotid artery of the lesion site. The intermediate catheter (ENVOY; Cerenovus, USA) was navigated to the lacerum segment of the internal carotid artery (ICA) to provide additional back-up support and followed by a 205-cm guidewire (Transcend EX soft tip, Boston Scientific, Costa Rica). The 205-cm guidewire was replaced with a 300-cm guidewire (Transcend EX soft tip, Boston Scientific, Costa Rica). Subsequent pre-dilation procedure (Emerge; Boston Scientific, USA) and wingspan stent (Stryker, Ireland) deployment were done in every patient. During pre-dilation, the balloon was inflated with a 2-atm per minute rate and monitored to ensure the pressure did not exceed 6 atm. The diameter of the balloon was chosen to accomplish dilatation of the lesion no more than 80–100% of the diameter of the parent artery. The diameter of the stent was chosen to be larger than the parent artery with an addition of 0.5–1 mm. Stent length was chosen with 3 mm in addition to the edge of the lesion. After the stent was implanted, post-dilation was done in the post-dilation group (Emerge; Boston Scientific, USA). Post-dilation balloon was targeted with an 80–100% size of the vessel. The size of the post-dilation balloon was the same as the pre-dilation one, but a new balloon catheter was used to facilitate catheter navigation. The rate of post-dilation balloon inflation was the same as the pre-dilation one. The stent was placed entirely by one experienced neurosurgeon (C.Y. Huang). A suture-mediated system (Proglide; Abbott, USA) was used for the puncture-site hemostasis.

### Clinical and Angiographic Follow-Up

The following parameters were collected: age, sex, smoking habits, body mass index, history of hypertension, diabetes mellitus, hyperlipidemia, the Modified Rankin Scale (mRS) upon admission and 3, 6, 12 months after the procedure, the National Institutes of Health Stroke Scale (NIHSS) score upon admission and after the surgery, clinical symptoms, the antithrombotic used upon admission, the time interval from stroke or TIA event to intervention, and neutrophil–lymphocyte ratio (NL ratio). The lesion length, Mori type, the extent of the stenosis, the diameter of parent vessels, the diameter of stenosis lumen, and the diameter of the stent were decided using digital subtraction angiography (DSA). The stenosis percentage and diameter of stent and lumen were measured before and immediately after the surgery and 1-year follow-up. We use Mori classification for lesion grouping (17). Periprocedural stroke and TIA were defined as new-onset neurological deficits within 30 days of the procedure. ISR was defined as stenosis >50% of the lumen.

### Statistical Analysis

Categorical variables were compared between groups using the chi-square test or Fisher's exact test. Continuous variables were presented as mean ± one standard deviation if normal distribution, otherwise as median with interquartile range. Independent Student *t*-test and Whitney *U*-test were used to compare the difference between the two groups. When comparing the stent and lumen size difference, paired *t*-test and repeated measurement analysis of variance (ANOVA) were utilized. The results were both sided, and the *p* < 0.05 was defined as a statistically significant result. We use the software GraphPad Prism 6.0 for calculation and figure construction.

## Results

### Basic Demographics and Complication Rate

A total of 28 patients underwent wingspan stenting revascularization during the study period (Table 1). Among them, 13 patients underwent no post-dilation stenting procedure, and the other 15 patients underwent post-dilation stenting surgery. The median age was 56 (47–71) years in the no post-dilation group and 67 (57–70) years in the post-dilation group (*p* = 0.22). Other variables which included sex, body mass index, history of smoking, diabetes mellitus, hypertension, hyperlipidemia, NL ratio, mRS and NIHSS before and after the procedure, antithrombotic agents upon admission, and duration from the stroke or TIA event to the procedure did not show a statistical difference between the two groups. Lesion-specific parameters including lesion site, lesion length, and Mori type also showed no difference between groups. The periprocedural complication rate in the whole cohort was 3.57%, attributed to one patient in the post-dilation group. The patient had critical basilar artery stenosis (85.3%) and suffered from a periprocedural complication with perforator stroke. No hemorrhage nor artery dissection was found. All our cases were asymptomatic during 1-year follow up except one patient in the post-dilation group who had lesion area-related stroke 9 months after the procedure. However, this patient had no ISR at the 1-year follow-up DSA. No stent dislocation was observed in the post-dilation group.

**Table 1:** Basic demographics for the no post-dilation and post-dilation group.

		**No post-dilation**	**Post-dilation**	
		***n* = 13**	***n* = 15**	***p***
Age, median (IQR)		56 (47–71)	67 (58–69)	0.21
Sex, *n* (%)	Male	9 (69%)	11 (73%)	>0.99
	Female	4 (31%)	4 (27%)	
Body mass index (mean ± SD)		25.25 ± 3.59	25.37 ± 3.68	0.94
Smoking, *n* (%)		4 (31%)	3 (20%)	0.67
Diabetes mellitus history, *n* (%)		4 (31%)	6 (40%)	0.71
Hypertension history, *n* (%)		9 (69%)	13 (87%)	0.37
Hyperlipidemia history, *n* (%)		10 (77%)	12 (80%)	>0.99
Neutrophil–lymphocyte ratio, median (IQR)		2.36 (1.53–3.06)	2.55 (2.30–5.70)	0.13
Events, *n* (%)	Stroke	10 (77%)	13 (87%)	0.64
	Transient ischemic attack	3 (23%)	2 (13%)	
Modified Rankin Scale, median (IQR)	Upon admission	1 (1–2.5)	1 (1–4)	0.86
	3 months	1 (0.5–1.5)	1 (0–2)	0.87
	6 months	1 (0.5–1.5)	1 (0–2)	0.87
	1 year	1 (0.5–1.5)	1 (0–2)	0.87
National Institutes of Health Stroke Scale, median (IQR)	Upon admission	1(0–2.5)	2 (1–5)	0.22
	After surgery	0 (0–1)	1 (0–3)	0.27
Medication upon admission, *n* (%)	Aspirin	10 (77%)	12 (80%)	>0.99
	Clopidogrel	7 (54%)	12 (80%)	0.23
	Statins	9 (69%)	8 (53%)	0.46
	Dabigatran	0 (0%)	1 (7%)	>0.99
	Cilostazol	2 (15%)	2 (13%)	>0.99
Time from event to surgery (days), median (IQR)		71 (47.5–183.5)	83 (34–178)	0.66
Lesion site, *n* (%)	Middle cerebral artery	8 (62%)	8 (53%)	>0.99
	Distal internal carotid artery	3 (23%)	4 (27%)	
	Vertebral artery	2 (15%)	2 (13%)	
	Basilar artery	0 (0%)	1 (7%)	
Mori classification, *n* (%)	A	3 (24%)	1 (7%)	0.52
	B	5 (38%)	8 (53%)	
	C	5 (38%)	6 (40%)	
Periprocedural complication, *n* (%)		0 (0%)	1 (7%)	>0.99
1 year follow up new events, *n* (%)		0 (0%)	1 (7%)	>0.99

*IQR, interquartile range; SD, standard deviation*.

### Comparison of Vessel Diameter and In-stent Restenosis Rate

The reference vessel diameter, the severity of ISR, and ISR incidence are recorded in Table 2. The reference vessel diameter for the middle cerebral artery in the no post-dilation and post-dilation groups was 2.91 ± 0.43 and 2.91 ± 0.78 mm, respectively (*p* = 0.99). Regarding the internal carotid artery and vertebrobasilar artery, the reference vessel diameter was 3.50 ± 0.62 and 3.84 ± 0.75 mm for each group (*p* = 0.43). The extent of stenosis before the surgery did not show a statistical difference (*p* = 0.60). When comparing the no post-dilation group and post-dilation group at the time when the stent was just deployed before post-dilation angioplasty, the stenosis rate was 28.5 ± 14.5% and 28.4 ± 14.6%, respectively (*p* = 0.99). However, after the post-dilation angioplasty, the stenosis rate was the same in the no post-dilation group, but 15.4 ± 10.1% in the post-dilation group (*p* < 0.01). After 1-year follow-up, the stenosis site in the no post-dilation group and post-dilation group were 50.1 ± 23.2% and 25.8 ± 17.5%, respectively (*p* < 0.01). The ISR rate was 39% in the no post-dilation group and 7% in the post-dilation group after a 1-year follow-up (*p* = 0.069).

**Table 2:** The lesion characteristics and the stenosis rate difference.

	**No post-dilation**	**Post-dilation**	
	***n* = 13**	***n* = 15**	***p***
**Reference vessel diameter (mm)**			
Middle cerebral artery (mean ± SD)	2.91 ± 0.43	2.91 ± 0.78	>0.99
Internal carotid artery and vertebrobasilar artery (mean ± SD)	3.5 ± 0.62	3.8 ± 0.75	0.43
**Lesion length (mm) (mean** **±** **SD)**	8.1 ± 4.5	7.9 ± 4.5	0.95
**Stenosis rate (%)**			
Preoperation (mean ± SD)	74.7 ± 8.80%	76.6 ± 10.2%	0.60
Post stenting (mean ± SD)	28.5 ± 14.50%	28.4 ± 14.6%	0.99
Post dilation (mean ± SD)	28.5 ± 14.50%	15.4 ± 10.1%	0.010
1-year follow-up (mean ± SD)	50.1 ± 23.2%	25.8 ± 17.5%	0.004
**In-stent restenosis incidence (%)**	38.50%	6.70%	0.07

*SD, standard deviation*.

### Stent Diameter and Lumen Diameter Following Time

**Figure 1:**
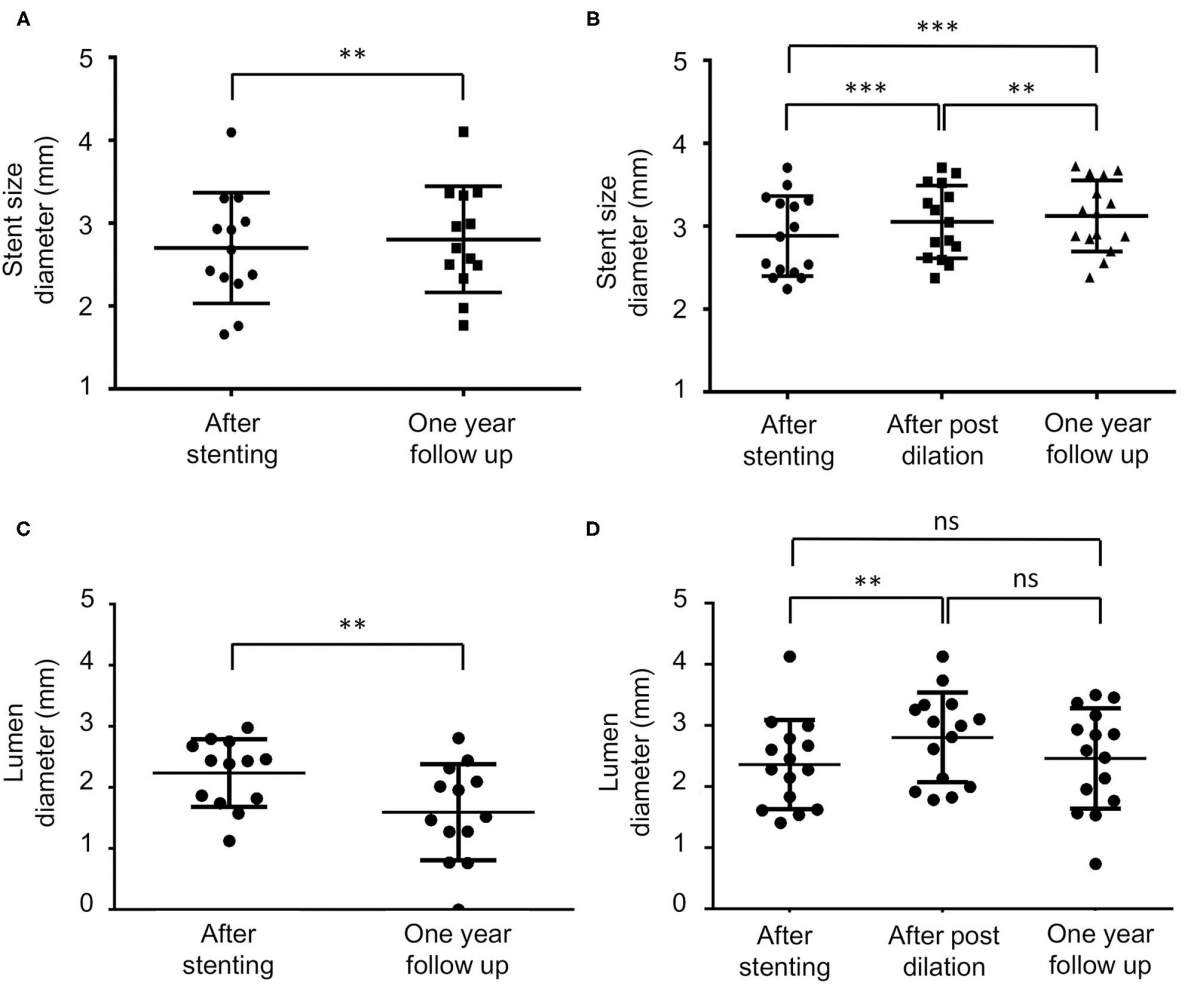
The stent and lumen diameter measurement during the procedure and at 1-year follow-up. ns, non-significant, **p < 0.01, ***p < 0.001. **(A,B)** Are the mean stent size in the no post-dilation and post-dilation groups, respectively. In the no post-dilation group, the diameter has a significant increase at a 1-year follow-up (2.70 ± 0.67 vs. 2.80 ± 0.64 mm *p* < 0.01). In the post-dilation group, the stent immediately increased after the post-dilation, and also at a 1-year follow-up (2.89 ± 0.48, 3.05 ± 0.44, and 3.12 ± 0.43 mm, *p* < 0.001, *p* < 0.01, *p* < 0.001). **(C,D)** Are the mean stenosis lumen diameter in the no post-dilation and the post-dilation groups, respectively. In the no post-dilation group, the lumen diameter significantly decreases at a 1-year follow-up (*p* < 0.01). In the post-dilation group, immediately after the post-dilation angioplasty, the lumen increased (2.36 ± 0.73 vs. 2.81 ± 0.73 mm, *p* < 0.01). At a 1-year follow-up, the lumen size decreased but did not significantly differ compared with the time when stent was just deployed and immediately after post-dilation (2.36 ± 0.73 vs. 2.46 ± 0.82 mm and 2.81 ± 0.73 mm, *p* = 0.88 and 0.17).

The stent size diameter measured immediately after the stent placement in the no post-dilation and post-dilation groups did not significantly differ (*p* = 0.50). Paired *t*-test and repeated ANOVA were used to compare the stent size diameter difference during the procedure and follow-up (Figure 1). In the no post-dilation group, the stent size diameter was 2.70 ± 0.67 mm after the stent was implanted, and 2.80 ± 0.64 mm at 1-year angiography (*p* < 0.01). In the post-dilation group, the mean stent size diameter was 2.89 ± 0.48, 3.05 ± 0.44, and 3.12 ± 0.43 mm when the stent was just placed, after post-dilation, and at 1-year follow-up. A significant difference was noted between the different timing in the post-dilation group (*p* < 0.001, *p* < 0.01, and *p* < 0.001). The mean lumen diameter for the no post-dilation group showed a decrease at 1-year follow-up (2.24 ± 0.56 vs. 1.60 ± 0.79 mm, *p* < 0.01). In the post-dilation group, though the lumen diameter increased immediately after balloon angioplasty (2.36 ± 0.73 vs. 2.81 ± 0.73 mm, *p* < 0.01), the lumen at 1-year follow-up decreased and did not have statistical difference with that before post-dilation (2.36 ± 0.73 vs. 2.46 ± 0.82 mm, *p* = 0.88).

## Discussion

A growing volume of literature substantiates endovascular treatment as a therapeutic option for ICAD. However, the high rate of ISR is still an obstacle that needs to be solved. We included properly selected patients with >70% stenosis treated with a wingspan system in the current study. Using a post-dilation method, we significantly reduced the incomplete apposition and expanded the wingspan system, which might help avoid in-stent restenosis at a 1-year follow-up. In addition, the periprocedural complication rate of the patients in the post-dilation group did not elevate compared with those without post-dilation. It was also comparable with a recent study (18).

**Figure 2:**
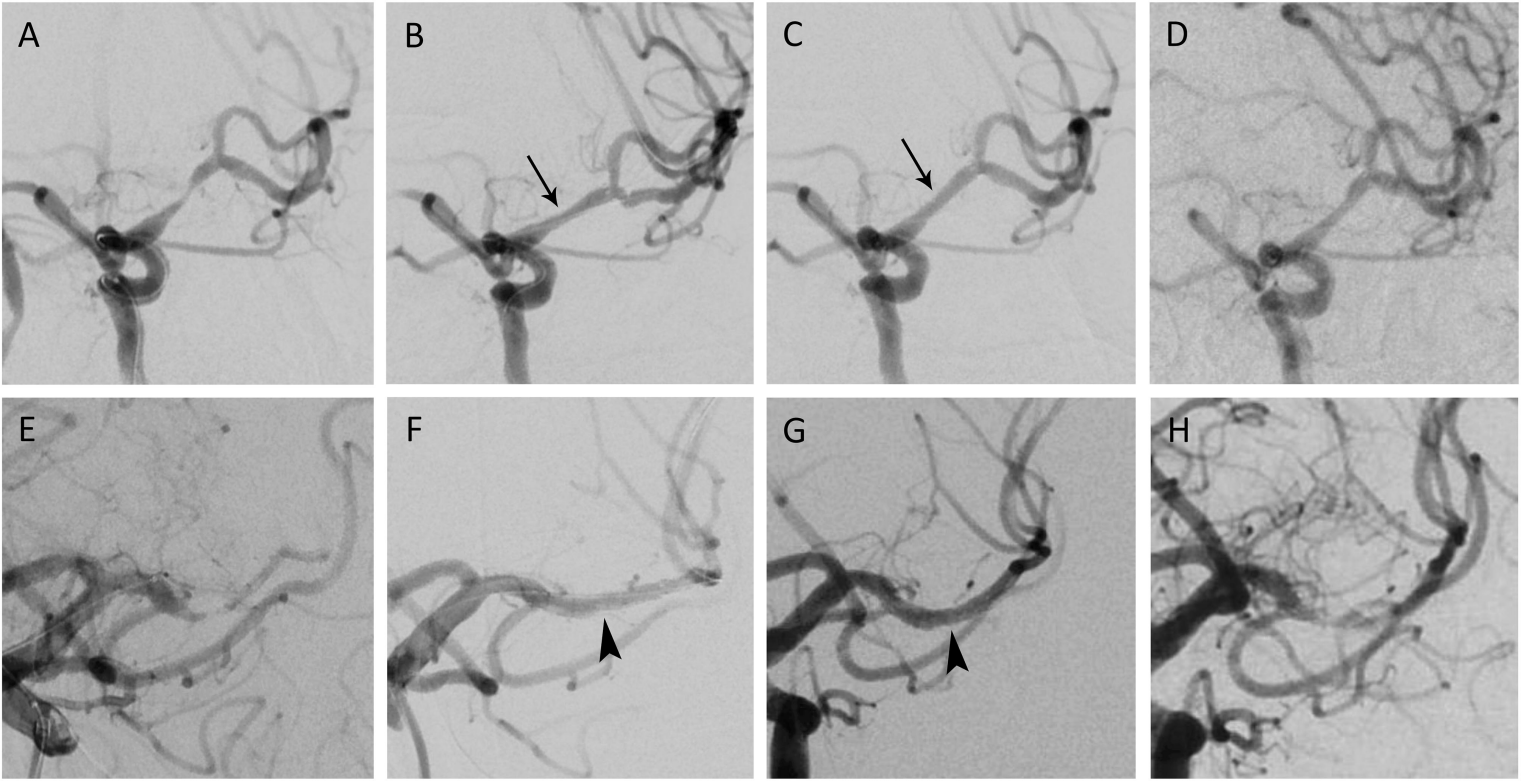
Percutaneous transluminal angioplasty and wingspan stenting of middle cerebral artery (MCA) stenosis. **(A)** A patient in their 60s presented with right hemiparesis due to cerebral infarction. The angiography showed left MCA stenosis by 79.7%, with a total of 5.5 mm in length. **(B)** After pre-dilation and wingspan stenting, the angiogram demonstrated 41.1% residual stenosis. **(C)** Post dilation decreased the residual stenosis to 16% (black arrow). **(D)** The 1-year angiographic follow-up showed stenosis by 32.1%. **(E)** Another patient in their 70s came for slurred speech after ischemic stroke. Angiography depicted 81.6% stenosis in the left MCA M1 segment; the lesion was 4.6 mm and eccentric. **(F)** After the wingspan stenting, the immediate residual stenosis was 14.6%. **(G)** Stent apposition improved after post stenting dilation (arrowhead), with residual stenosis downsized to 10.7%. **(H)** After 1 year, 77.7% of multifocal in-stent restenosis was observed. The patient was asymptomatic, with a stationary Modified Rankin Scale.

The ISR rate in previous studies was 14–29.7% (11, 19), but when utilizing the post-dilation method, the ISR rate had decreased to 6.67% in our study. Though the exact mechanism of ISR in the intracranial artery had not been well-studied, it had been investigated in coronary and other arteries for decades. The possible causes of ISR included, stent under expansion, intimal hyperplasia, and neoatherosclerosis (20, 21). In a coronary artery study, the prevalence of stent under expansion was higher in the patient with ISR (21). Another study proved that suboptimal or incomplete stent expansion has worse outcomes in both short and long-term follow-up in a coronary artery (22). In addition, acute incomplete coronary stent apposition may increase the possibility of its persistence (23). Our results showed that post-dilation could be observed as the stent diameter significantly increased immediately after the post-dilation and persisted through a 1-year follow-up. The cerebral angiographies of two representative patients are shown in Figure 2. The extent of stenosis also had a significant difference between the post-dilation and no post-dilation groups. Because we used the same angioplasty pressure for pre-stenting dilation and post-stenting dilation, we believe that this resulted in a more optimal stent expansion and less stent recoil. Several studies proved that post-dilation can provide enough resistive outward force to achieve better stent expansion and apposition to reduce ISR, which is consistent with our results (24, 25).

Due to the self-expandable property of the wingspan stent, the diameter of the stent would increase with time after deployment. Our study showed that although stent size increased in both the no post-dilation and post-dilation groups in 1-year follow-up, the vessel lumen showed stationary or decreased in each group. Several studies that evaluated self-expandable stents in the carotid artery and coronary artery also illustrated that the stent size increased following time (26, 27). However, despite the gain in the stent size, the actual lumen in these studies had shown stationary or mildly decreased (26, 27) due to smooth muscle cell proliferation and neointimal hyperplasia, which were consistent with our results. A pilot study evaluated the lumen gain for the wingspan stent for 82.4 months and showed gradually enlarged lumen after stenting (28). The discrepancy might result from the different stent designs and different treatment protocols. Besides, the intracranial artery anatomy was different from an extracranial artery (3), which may result in different vessel wall reactions to the stent. Further studies were needed to delineate the exact molecular mechanisms of the ISR and the lumen gain in the wingspan system.

Regarding the safety of the post-dilation method, one of the patients in our study had periprocedural complications in the post-dilation group, leading to a complication rate of 6.7% in the post-dilation group and 3.6% in the whole cohort. This complication rate was inconsistent with recent PTAS studies (18) and lower than the SAMPPRIS and VISSIT trials. Previous studies on post-dilation in coronary arteries showed that aggressive post-dilation with balloons disproportionately larger than the vessel could lead to stent deformation and more neointimal proliferation (29). However, we used the same size of the balloon and the same inflation pressure for the pre-dilation and post-dilation with a reasonable target volume. In addition, Foin et al. inflated their balloon up to 14 atm, in contrast to our low pressure of 6 atm. Therefore, no stent-related complication or stent disfigurement was noted in our study. Since the ISR rate was much lower in the post-dilation group in the current study, the post-dilation group did not show a more neointimal formation trend.

There are some limitations of this study. First, though this was a pilot study, the results were generated by a single center. A further multicentric study will be warranted. Second, the timing of the post-dilation was started in December 2019. Therefore, the two groups were not equally balanced in the perspective of time. Third, we had limited follow-up intervals. Though most ISRs occurred within 1 year (30), longer follow-up data are still needed for long-term survival and neurological outcome analysis.

## Conclusion

With the improving image and surgery technique, the wingspan system is on the horizon as a treatment modality for ICAD. The appropriate method for prevention and treatment for ISR is still to be determined. In this study, we showed that post-dilation can be feasibly done and can prevent the ISR. Our study provided a practical method for an interventional neurologist to obviate ISR in the wingspan system.

## Data Availability Statement

The raw data supporting the conclusions of this article will be made available by the authors, without undue reservation.

## Ethics Statement

The studies involving human participants were reviewed and approved by Institutional Review Board of National Cheng Kung University Hospital. Written informed consent for participation was not required for this study in accordance with the national legislation and the institutional requirements.

## Author Contributions

C-YH and Y-TS conceptualized the study. P-SP and C-YH wrote the original draft. Y-HS, J-SL, L-CW, and C-YH reviewed and edited the manuscript. C-YH provided the supervision. All authors reviewed and approved final version of the manuscript.

## Conflict of Interest

The authors declare that the research was conducted in the absence of any commercial or financial relationships that could be construed as a potential conflict of interest.

## Publisher's Note

All claims expressed in this article are solely those of the authors and do not necessarily represent those of their affiliated organizations, or those of the publisher, the editors and the reviewers. Any product that may be evaluated in this article, or claim that may be made by its manufacturer, is not guaranteed or endorsed by the publisher.
